# Quantitative Comparison of Binary Mix of Agro-Industrial Pozzolanic Additions for Elaborating Ternary Cements: Kinetic Parameters

**DOI:** 10.3390/ma14112944

**Published:** 2021-05-29

**Authors:** Ernesto Villar-Cociña, Moisés Frías, Holmer Savastano, Loic Rodier, María Isabel Sánchez de Rojas, Isabel Fuencisla Sáez del Bosque, César Medina

**Affiliations:** 1 Department of Physics, Central University of Las Villas, Santa Clara 54830, Cuba; 2 Materials Recycling Department, Eduardo Torroja Institute (CSIC), c/Serrano Galvache, 4, 28033 Madrid, Spain; mfrias@ietcc.csic.es (M.F.); srojas@ietcc.csic.es (M.I.S.d.R.); 3 Research Nucleus on Materials for Biosystems, University of Sao Paulo, P.O. Box 23, Pirassununga 13635-900, SP, Brazil; holmersj@usp.br (H.S.); rodierloic@gmail.com (L.R.); 4 Department of Construction, Institute for Sustainable Regional Development (INTERRA), University of Extremadura, Avda. De la Universidad, s/n, 10003 Cáceres, Spain; isaezdelu@unex.es (I.F.S.d.B.); cmedinam@unex.es (C.M.)

**Keywords:** quantitative characterization, binary combinations, pozzolanic activity, kinetic parameters, kinetic-diffusive model, ternary cements

## Abstract

In this research work, the quantitative characterization of a binary blend comprised of two pozzolans (sugar cane straw (SCSA)–sugar cane bagasse ashes (SCBA), bamboo leaf ash (BLAsh)–SCBA and paper sludge (PS)–fly ash (FA)) taking into account the calculated values of the kinetic parameters of the reaction in the pozzolan/calcium hydroxide system is shown. The paper shows the most significant and important results obtained by the authors in the quantitative assessment (calculation of kinetic parameters) of the pozzolanic reaction of different mixtures of pozzolanic materials that are residues from agriculture or industrial processes. This allows a direct and rigorous comparison of the pozzolanic activity of the binary combinations of materials. The values of the kinetic parameters (reaction rate constant or activation free energy) constitute a very precise quantitative index of the pozzolanic activity of the binary combinations of materials, which is very useful for its employment in the elaboration of ternary cements. This paper shows that the binary blends 1SCBA60Blash40, 1SCBA50Blash50, 1SCBA70Blash30 have a very high pozzolanic reactivity followed by PSLSFA, 2SCBA50SCSA50, PSISFA and SCWI.

## 1. Introduction

The use of agro-industrial waste is currently a topic of great interest, the subject of many research works. Most of these materials contain high amounts of silica and/or alumina which allow them to be used as pozzolanic additions in commercial Portland cement. The hydrated phases that are formed during the pozzolanic reaction can greatly improve the performance of concrete [1,2,3,4,5,6,7,8].

The use of agricultural and industrial residues for the production of concrete is a widespread practice worldwide. The permanent and large-scale generation of waste materials has become a serious environmental problem due to the pollution they produce and the space they need for their disposal. This represents a serious problem for agricultural and industrial companies. On the other hand, the evaluation of the pozzolanic reactivity of pozzolanic materials for use as an additive material to cement has become increasingly important because it represents greater sustainability in cement production.

The vast majority of agro-industrial waste is placed in landfills and open spaces, causing a negative effect on the environment. The use of waste as pozzolanic additions to cement makes it possible to reduce these landfills; in addition, the consumption of clinker per ton of cement decreases with the incorporation of additions, which represents great energy saving as well as a decrease in the emission of CO_2_ to the atmosphere.

During the last decades, there has been a change in the research related to cement matrices. In general terms, it can be said that during the 20th century, most scientific-technical studies were focused on binary cements (with one added additive). However, during the last decade, research developments are focusing on innovative cements made with more than one admixture. This is demonstrated by the large number of scientific contributions that have appeared in the international literature in recent years. These contributions show the combination of three mostly standard products: fly ash–slag, silica fume–fly ash and silica fume–slag [9,10,11,12,13,14,15,16]. In all cases, better results are obtained with ternary cements compared to binary cements (type II CEM), both in pastes and mortars as in concrete. This scientific interest in ternary cements confirms the growing trend of their production in different countries. Given the current economic situation and the serious environmental problems in which we are involved, it is conceivable that this type of cement, with more than one pozzolan, will take the lead in future commercial cement manufacturing due to its lower production cost.

The possibility of the development and production of ternary cements in developing countries is limited, taking into account the limitation of large-scale use of standardized systems: fly ash–slag, silica fume–fly ash and silica fume–slag.

For this reason, in addition to the pozzolans traditionally used in international standards (fly ash, silica fume, natural and calcined pozzolan), there is also research aimed at finding new materials that may be included in these standards in the near future. For this, it is necessary to carry out pre-normative studies that establish the technical basis for the use of these new products and agro-industrial waste as materials with cementitious properties [17,18,19,20]. In the case of calcined agricultural residues, the research works that report their characterization and use in ternary cement matrices are scarce [17,18,19,20,21,22].

Also, the increasing interest in the utilization of ternary cements is a direct result of their excellent performance, which has been frequently attributed to the synergistic effects taking place between the pozzolanic materials in the binary blend. The methods used in most of these investigations to evaluate the pozzolanic activity of the materials and their binary combinations are essentially directed to the qualitative aspect of the behavior of the binary mixtures of materials, not the quantitative aspect of the pozzolanic reaction in the binary mixture/cal system, which focuses on calculating the kinetic parameters of this reaction. There are some methodologies for the calculation of the parameters in different systems. In our research work, we use the application of a mathematical model based on well-founded physical hypotheses and its fitting to the experimental data by non-linear methods, which allows us to determine the kinetic parameters in the fitting process of the model, other common methodologies for parameter estimation and identifiability analysis are employed, among which is the Bayesian approach. The use of the Bayesian method is also of great interest and acceptance in different areas, including the engineering field [23].

In our case, the experimental monitoring of the pozzolanic reaction kinetics allows knowing the constants that characterize the reaction rate. Knowing these constants, it is possible to evaluate the different types of pozzolanic materials for their effective application as additions in mortars and concretes; it is also possible to compare their properties.

Currently, the acceptance by the international scientific community of the use of kinetic coefficients as a rigorous criterion for evaluating the pozzolanic reactivity of materials is already a fact. With this objective, traditional mathematical models have been developed and applied to study the pozzolanic reaction kinetics [24,25,26,27,28,29,30,31], which describe the experimental results with greater or lesser precision. Other researchers [32,33,34,35] have continued the development of new models that allow the quantitative characterization of the reaction kinetics in pozzolan/CH and cement/CH systems. Recently, Villar-Cociña et al. [36,37,38] developed a new kinetic-diffusive model that allows calculating the kinetic parameters of the pozzolanic reaction in the sugarcane/CH waste system and quantitatively characterizing the pozzolanic activity of these materials for all reaction ages. A good correlation between the experimental data and the theoretical model was obtained, which motivated the application of the model to evaluate different pozzolans.

This paper shows important results obtained by the authors in the study and quantitative characterization of several binary pozzolanic systems: sugar cane bagasse ash (SCBA) and sugar cane straw ash (SCSA), SCBA and bamboo leaf ash (BLAsh), calcined paper sludge (CPS) and Fly Ash (FA) applying the kinetic-diffusive model, which allows the calculation of the kinetic parameters of the pozzolanic reaction.

This allowed the rigorous characterization (obtaining the kinetic parameters) of these binary mixtures of materials, as well as a direct comparison of the pozzolanic behavior of the binary systems, the dosages of its components and their origins (industrial or laboratory).

The above is very useful for the selection of one or another binary pozzolanic system, which will depend on the characteristics needed for the building site. For elaborating on ternary cements, which is of great importance in the actuality due to the present world-wide economic crisis, the knowledge of the kinetic parameters is a fundamental tool for quantifying the reactivity of the pozzolanic materials that will be added to the cement. Other complementary experimental techniques were also used in the research including scanning electron microscopy (SEM), X-ray diffraction (XRD), porosimetry, which are not shown in the article for reasons of space, this would lead to the article being too long and would not contribute to the essence of the subject.

## 2. Materials and Methods

### 2.1. Materials

The materials analyzed involve both artificial pozzolan (thermally activated waste) and natural pozzolans that are readily available in industrial and agricultural economies. For the selection of the calcining temperature necessary for the activation of the materials, the best results (high reactivity) from some research works were taken into account [17,18,39,40].

The samples and their designations are shown in Table 1.

#### 2.1.1. Binary System SCBA and SCSA

Two different agricultural ashes were employed: (i) an industrial bottom ash, a blend of SCBA and SCSA (SCWI), from a Brazilian sugar cane mill, used as biomass for cogeneration (around 40–50% of the mix, reaching 700–800 °C in the burning process); and (ii) ashes produced in the laboratory (2SCBA50SCSA50) resulting from a 1:1 ratio (by weight) of sugar cane bagasse and sugar cane leaf ashes, separately burned in an electric furnace (heating ramp of 10 °C/min up to 700 °C for 90 min).

Both ashes were ground and sized down to below 90 μm, similar to the commercial Portland cement [41].

#### 2.1.2. Binary System SCBA and BLAsh

The raw materials were collected in the Pirassununga region, state of Sao Paulo, Brazil: bamboo leaves from the University of Sao Paulo Campus and sugar cane bagasse furnished by the Abengoa industry.

The raw materials were burned in a muffle furnace (JUNG 10010, Brazil) at 400 °C for 60 min and then, calcined at 700 °C for 60 min for sugar cane bagasse [42] and 600 °C for 60 min for bamboo leaves [21,43] (10 °C/min of heating ramp). The pozzolanic activity of the resulting ashes is the highest, for those specific temperatures. Fast cooling from the muffle furnace to room temperature was adopted to obtain an amorphous structure. The ashes were then ground and sieved until below 90 µm [9].

#### 2.1.3. Binary System PS and FA

Two paper sludges were used: (i) a Spanish paper sludge activated at 700 °C for 2 h on laboratory scale (LS), according to [39,44]; (ii) a paper sludge waste activated in a fluidized bed system for the generation of electric energy. The industrial process operates in the range of 720–740 °C and the ash particles are collected by a filtering system (IS). The patented pozzolanic ashes were marketed as ‘‘Topcrete’’, CDEM [45].

Fly ash (FA) from Spain was utilized as a traditional pozzolan for the Portland cement industry [46]. At the first step, each pozzolan was ground and sieved at 90 μm. After, the mixes LS/FA and IS/FA were prepared at proportions of 50/50 (by weight) [47].

#### 2.1.4. Lime

Calcium hydroxide (CH) (95% of minimum purity, 4.8% of maximum magnesium and alkaline salts content and 0.5% of maximum insoluble substance content) was used in the pozzolans/lime suspension.

To differentiate the pozzolans in the binary mixture that are the most abundant (1SCBA, 2SCBA and PS), we will call them base pozzolans. Those that are less abundant and complement the binary mixture will be called complementary pozzolans.

### 2.2. Test Methodologies

#### 2.2.1. Pozzolanic Activity Methods

To carry out a qualitative or quantitative determination of pozzolanic activity many experimental methodologies have been developed [48,49,50].

In this research, as in other studies carried out by the authors [39,40,51,52], two different pozzolanic activity methods were applied. Both methods are based on the monitoring of the lime consumption with the reaction time and they can be used indistinctly.

It is important to note that the application of the kinetic-diffusive model used in this research to calculate the kinetic parameters of the pozzolanic reaction is independent of the method used for the evaluation of the pozzolanic activity, these have been shown elsewhere [51].

(a) Conductometric method

This method follows the conductivity of the pozzolan/calcium hydroxide solution with reaction time.

A saturated solution of calcium hydroxide (CH) was prepared, for which deionized water and CH were used. Both were mixed and the solution was stirred for 2 h, then the solution was kept at rest for 24 h, it was subsequently filtered and evaluated with hydrochloric acid.

Each 100 mL of saturated Ca(OH)_2_ solution was mixed with 2.10 g of pozzolanic material (which is the proportion commonly found in the literature for similar experiments) and magnetically stirred. Immediately after the pozzolan was mixed with the CH solution, the conductivity measurements began.

The measurements of conductivity were made at (40 ± 1) °C at different times. To correlate the CH concentration with the conductivity of the CH solution, a calibration curve that demonstrates a linear dependence was applied [19,36,53].

(b) Accelerated chemical method

This method also follows the pozzolan-calcium hydroxide reaction over time. The test consisted of putting the pozzolanic material (1 g) in contact with a saturated lime solution (75 mL) at (40 ± 1) °C for 1, 7, 28 and 90 days. At the end of each period, the CaO (quicklime) concentration in the solution was analyzed. Fixed calcium hydroxide (mmol/L) was obtained as the difference between the original concentration of a solution of saturated calcium hydroxide and the CaO found in the solution in contact with the sample, at the end of a given period [52].

#### 2.2.2. Mathematical Model

It is known that the pozzolanic reaction in CH/pozzolan systems is heterogeneous and it can be considered a solid-solution type reaction:

A(L) + bB(S) → F(S) + E(L)(1)

For elaborating on the model, we initially used the Decreasing Nucleus Model (DNM). In accordance with this model, when solution A reacts on the surface of reactant B (stoichiometry coefficient b) a reaction product layer F is formed around the unreacted nucleus of reactant B which gradually reduces. The diffusion of A through layer F (in the case this is porous) occurs until it reaches the interface between F and the unreacted nucleus. E(L) refers to the fluid products of the solid-solution reaction.

The following assumptions were made:

− The spherical form of the granule is retained, and the densities of F and B are the same. Consequently, the total radius of the granule *r_s_* (considering the reaction product layer and the nucleus without reacting) does not change with time and an intermediate region does not exist between the nucleus and the layer of product [54].
− The movement rate of the reaction interface, dr_c_/dt, is small in comparison to the diffusion speed of A through the product layer (pseudo-stable state) [55]. This is valid when the density of the fluid in the pores of F is smaller than the density of the solid reactant, which is certain in general.
− Taking into account the pseudo-stable conditions, where the speed equations, expressed as a mole of solution A (CH solution) that disappears per unit of time per particle, are identical, the rate equation is obtained by handling these equations and considering well-founded physical hypotheses. It is determined by the control regime or by the rate-limiting step which can be [56,57]: (i) diffusive control and (ii) kinetic control.
− In accordance with all the above, Villar-Cociña et al. [36,37] proposed a kinetic-diffusive model that allows the characterization of the pozzolanic activity in the sugar cane straw-clay ash/CH solution. Subsequently, the model was perfected in the characterization of the reaction kinetics in the sugar cane straw ash/CH and sugar cane bagasse ash/CH systems, where a correction term *C_corr_* was incorporated. This term is related to the remaining concentration of CH, which in some systems is not consumed totally. The corrected model is [38]:

(2) $$\xi =\frac{C_{0}-C_{t}}{C_{0}}=1-\frac{0,23.Exp\left(\frac{-3\;t}{\tau }\right).\left(-1+\;Exp\left(\frac{t}{\tau }\right)\right).\frac{1}{\tau }}{C_{o}\;D_{e}\;r_{s}}+\frac{0,23.Exp\left(-\frac{t}{\tau }\right).\frac{1}{\tau }}{C_{0}\;K\;{r_{s}}^{2}}-C_{corr}$$

where: *D_e_* is the effective diffusion coefficient of A through the porous layer of product F; K is the reaction rate constant; *C_o_* is the initial concentration of the solution; and *τ* is a constant of time (time interval in which the radius of the nucleus of pozzolan diminish to 37% of its initial radius (*r_s_*)). The radius *r_s_* of the pozzolan particles was taken as the average size particle for each analyzed material.

The dimensionless magnitude *ξ* = (*C*_0_ − *C_t_*)/*C*_0_ represents the relative loss of lime concentration and *C_t_* represents the absolute loss of lime concentration with time for the pozzolan/CH system.

It is well known that the pozzolanic reaction develops in stages. Therefore it may happen that the rate-limiting stage corresponds with the diffusion through the layer of reacting product or with the chemical reaction at the surface of the unreacted nucleus.

Consequently, it is possible to have different behavior: diffusive (described by the second term of Equation (2)), kinetic (third term) and kinetic-diffusive (second and third terms). Further explanations of the model can be found elsewhere [36,37].

The fitting process of this model allows the computing of the kinetic parameters (effective diffusion coefficient and reaction rate constant) and, therefore, a rigorous characterization of the process.

## 3. Results

### 3.1. Chemical Characterization

The chemical compositions of the samples were determined by an X-ray fluorescence (XRF) technique (Table 2).

#### 3.1.1. SCBA/BLAsh System

Silica (SiO_2_) is the major constituent followed by K_2_O, Al_2_O_3_, Fe_2_O_3_, CaO, P_2_O_5_, MgO, SO_3_ and TiO_2_ in the sugar cane bagasse ashes (1SCBA). On the other hand, the major component is SiO_2_ followed by CaO, K_2_O, SO_3_, MgO and P_2_O_5_ in bamboo leaf ashes (BLAsh).

The presence of amorphous silica is due to the absorption of the silicic acid from the soil followed by the deposition in the different parts of the plant [58]. The sums of SiO_2_ + Al_2_O_3_ + Fe_2_O_3_ for 1SCBA and BLAsh are 57% and 72%, respectively. The chemical composition of 1SCBA and BLAsh differs from each other due to the grass precursors, the plant maturity and the soil where they are grown. 1SCBA presented the highest content of K_2_O (13% by mass); K_2_O is associated mainly with the fertilizers utilized in the sugarcane fields. Some authors demonstrated the use of sugarcane bagasse ashes with high K_2_O content (9 wt%) as mineral addition in cement-based binders [59,60].

The account SiO_2_ + Al_2_O_3_ + Fe_2_O_3_ for the two ashes is higher than 50% (minimum amount recommended by ASTM C618 [61]), being considered as a pozzolanic material for partial cement replacement. The loss on ignition (LOI) of all ashes is below 10 wt%, indicating that the calcination was efficient to remove organic and volatile compounds.

#### 3.1.2. SCBA/SCSA System

Table 2 shows the chemical composition of the binary mix 2SCBA50SCSA50 and SCWI. These two ashes are similar but with different amounts of the main oxides. The laboratory ashes (2SCBA50SCSA50) showed a silico-calcium nature and the industrial ashes (SCWI) presented a silico-alumina-ferrous nature. The chemical composition revealed low silica content (50–60%) in comparison to other bagasse ashes (65–80%) [62], although the corresponding values were similar for sugar cane ash from the cogeneration process (58%) available in the international literature [63].

In both ashes, K_2_O was in the range between 6.0 and 8.5%, similar to other agro-industrial ashes reported in the literature [64]. LOI values were below 10%. According to Chusilp et al. [65], the referred composition will generate a very reactive pozzolan.

The compounds of the mixes: The main oxide present in SCSA and 2SCBA ashes is SiO_2_, followed by CaO and Al_2_O_3_ respectively. Other oxides such as MgO, SO_3_, K_2_O and P_2_O_5_ are present in smaller amounts. The LOI of the ashes is under 10% (by mass), indicating that the burning conditions were enough for the removal of the organic and volatile compounds.

#### 3.1.3. Paper Sludge/FA System

The chemical results (by XRF) of the three pozzolans follow in Table 2. Based on the analytical data, it is possible to observe that the two starting sludges were formed by SiO_2_, Al_2_O_3_ and CaO, accounting for more than 69% of the total mass. For FA, the sum of SiO_2_, Al_2_O_3_ and Fe_2_O_3_ represents 85% of the total mass. The activated paper sludges showed LOI above 20%, which can be understood to be due to the calcite present in the starting materials, as detected by the XRD patterns [47].

### 3.2. Pozzolanic Activity

**Figure 1:**
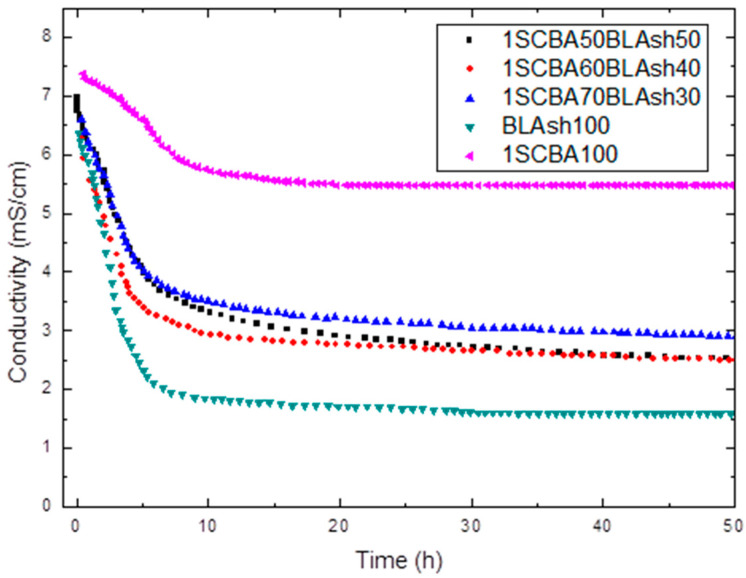
Variation of electrical conductivity with reaction time for binary system 1SCBA + BLAsh.

**Figure 2:**
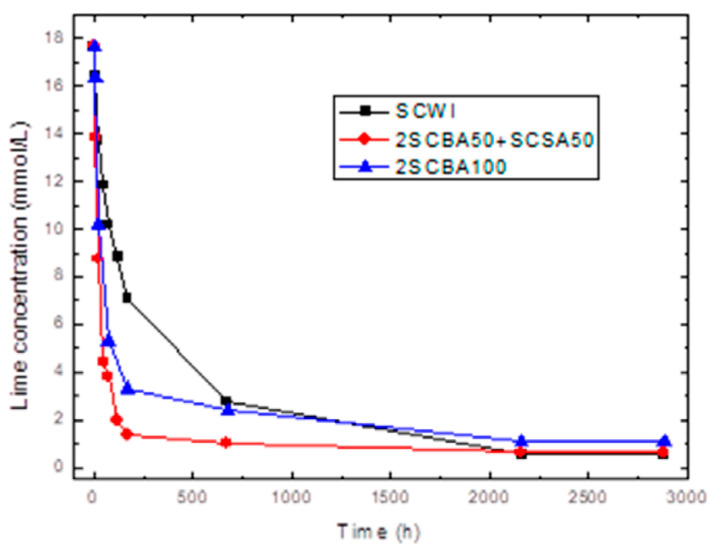
Variation of electrical conductivity with reaction time for the binary system 2SCBA + SCSA.

**Figure 3:**
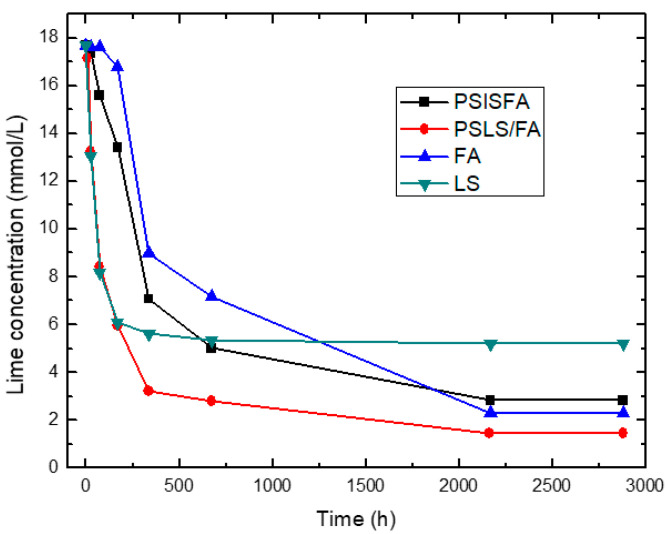
Variation of electrical conductivity with reaction time for the binary system PSIS + FA, PSLS + FA.

The results obtained for pozzolanic activity are shown in Figure 1, Figure 2 and Figure 3. In Figure 1 the conductivity variations (milliSiemens per centimeter) versus time (in hours) for the pozzolans/CH suspensions are shown for the binary mixtures of pozzolans 1SCSA50Blash50, 1SCBA60BLAsh40, 1SCBA70BLAsh30 and the pozzolans 1SCBA100 and BLAsh100; while Figure 2; Figure 3 show the results of accelerated pozzolanic tests for SCWI, 2SCBA50SCSA50CSCA, 2SCSA100 and PSISFA, PSLSFA, FA, LS respectively, ones up to 120 days of reaction. In the present study, there was no influence of the pozzolanic activity methods on the computing of the kinetic parameters [51].

In the conductometric test (Figure 1) a decrease in the electrical conductivity of the solution is obtained. The formation of hydrated phases with the corresponding decrease of the CH concentration in the solution (which leads to a decrease in conductivity) can be the cause of this behavior.

A considerable loss of conductivity in early ages is obtained; is evident that BLAsh consumes more CH than 1SCBA. From the qualitative point of view, the high reactivity of BLAsh, which is comparable to silica fume (SF) [66] (a highly expensive pozzolan) is appreciated, followed by 1SCBA60BLAsh40, 1SCSA50Blash50 and 1SCBA70BLAsh30. The 1SCBA shows the lowest consumption of CH (minor reactivity).

The stabilization of the curve is more rapidly reached for BLAsh and the binary mixtures in comparison with 1SCBA. The stabilization of the curve indicates the moment when the reaction has finished practically.

However, it is very important to take into account another interesting factor when an evaluation of the pozzolanic reactivity is carried out. This is the difference between the initial and final conductivities (stabilized conductivity) in the different pozzolan/CH solutions. To a greater difference, the reactivity of the material will be higher [67]. This could be related to calcium hydroxide consumption, a greater difference corresponds to greater consumption of CH in comparison with a minor difference.

For example, for the binary pozzolanic mixture 1SCBA + BLAsh, a qualitative analysis shows a greater reactivity for 1SCBA60BLA40 followed by 1SCBA50BLA50 and 1SCBA70BLA30. The 1SCBA100 shows the lowest reactivity. The sample BLAsh100 has a much higher fixation of calcium hydroxide than 1SCBA100.

The result of the binary mixtures is very important in comparison with the 1SCBA100 sample, this implies that with fewer amounts of BLAsh (which are abundant in a smaller quantity), higher reactivities can be obtained than with 1SCBA100 which is very abundant, that is, the combination of both offers better qualitative results than if we use bagasse alone, a good synergy effect.

Theoretically, the higher content of silica is, the higher the fixation of calcium hydroxide. However, 1SCBA60BLAsh40 has a higher fixation of calcium hydroxide than 1SCBA50BLAsh50. This result can be explained by the fact that SCBA contains the finest particles compared to BLAsh, which causes an increase in the fixation of calcium hydroxide.

However, for the case of the materials shown in Figure 1, it is evident that 1SCBA60BLAsh50 consumes more CH than the 1SCBA100 but does not provide information on the rapidity of the reaction. The difficulties of the qualitative analyses are evident, for this reason, it is very important to evaluate from the quantitative point of view of the pozzolanic reactivity. This involves all these aspects and allows the computation of the kinetic parameters.

On the other hand, the results of accelerated pozzolanic tests for reaction times of up to 90 days are shown in Figure 2 and Figure 3. The absolute loss of lime concentration (CH) plotted against the reaction time for the pozzolans/CH samples is shown.

According to Figure 2, a considerable loss of lime concentration in early ages is obtained for the samples 2SCBA50SCSA50 and 2SCBA100. The stabilization of the curve was reached after a long period of time and it depends on the analyzed sample. A qualitative analysis shows a greater reactivity for 2SCBA50SCSA50 followed by 2SCBA100 and SCWI. The result of the binary mixture 2SCBA50SCSA50 in comparison with the sample 2SCBA100 is very important; this implies that with fewer amounts of SCSA (which are abundant in a lower quantity than sugar cane bagasse) higher reactivities can be obtained. The combination of both offers better qualitative results than if we used bagasse alone, a good synergy effect. According to Figure 3, a considerable loss of lime concentration in early ages is obtained for the samples PSLSFA and LS. The stabilization of the curve was reached after a long period of time and it depends on the analyzed sample. A qualitative analysis shows a greater reactivity for the mixture PSFSFA followed by LS. The mixture PSISFA and FA show a minor reactivity.

We must point out the result of the binary mixture PSLSFA in comparison with the samples LS and FA, it implies that the combination of both offers better qualitative results than if we use FA or LS only.

### 3.3. Application of the Mathematical Model and Determination of the Kinetic Parameters

As mentioned above, the knowledge of the kinetic parameters of the pozzolanic reaction is a good criterion for evaluating quantitatively the pozzolanic activity of the materials and their combinations. In the international bibliography, the values reported of kinetic parameters for pozzolanic binary mixes/CH solution are very scarce. We have not encountered in the literature at our disposal any attempt reporting on the kinetic parameters for the combinations of pozzolanic materials that allow the quantitative comparison of their reactivity.

With the aim of computing the kinetic parameters, the kinetic-diffusive model (Equation (2)) was applied for all samples.

**Figure 4:**
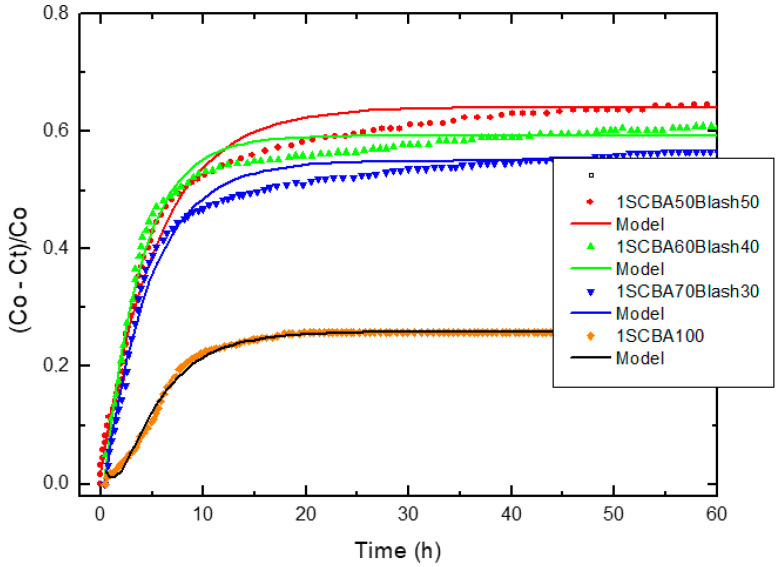
Relative loss of lime concentration versus reaction times for 1SCBA50BLAsh50, 1SCBA60BLAsh40, 1SCBA70BLAsh30 and 1SCBA100. (**Note**: Black circle (experimental), solid line (model)).

**Figure 5:**
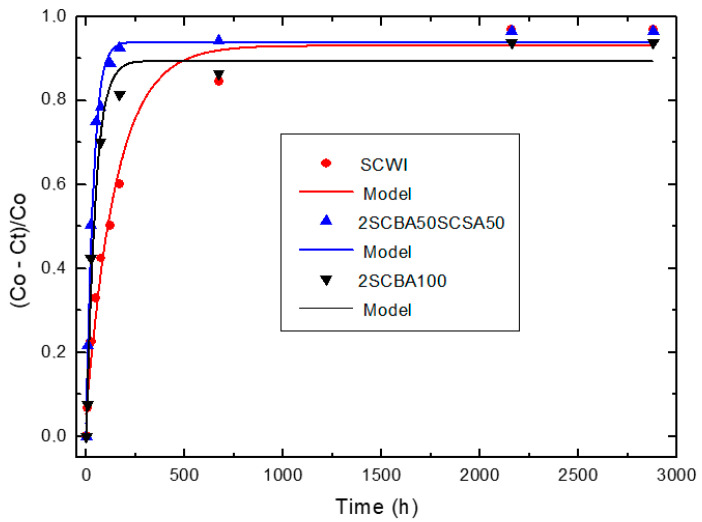
Relative loss of lime concentration versus reaction times for SCWI, 2SCBA50SCSA50 and 2SCBA100 (**Note**: Black circle (experimental), solid line (model)).

**Figure 6:**
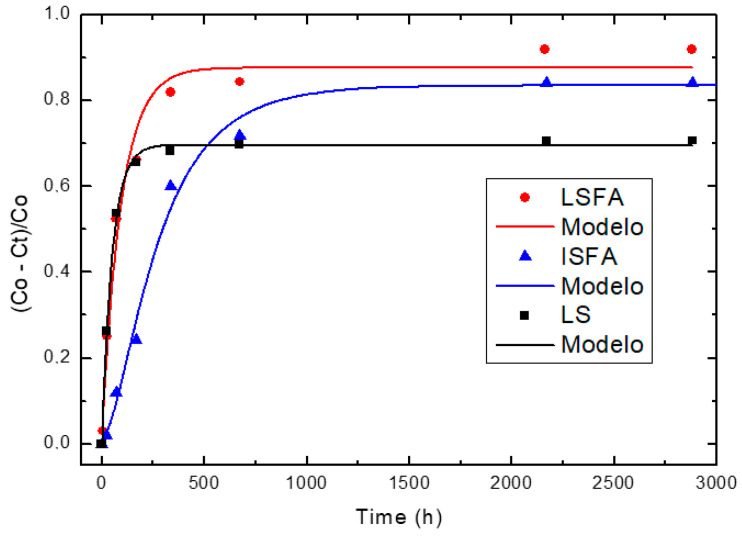
Relative loss of lime concentration versus reaction times for PSLSFA, PSISFA and LS (**Note:** Black circle (experimental), solid line (model)).

**Figure 7:**
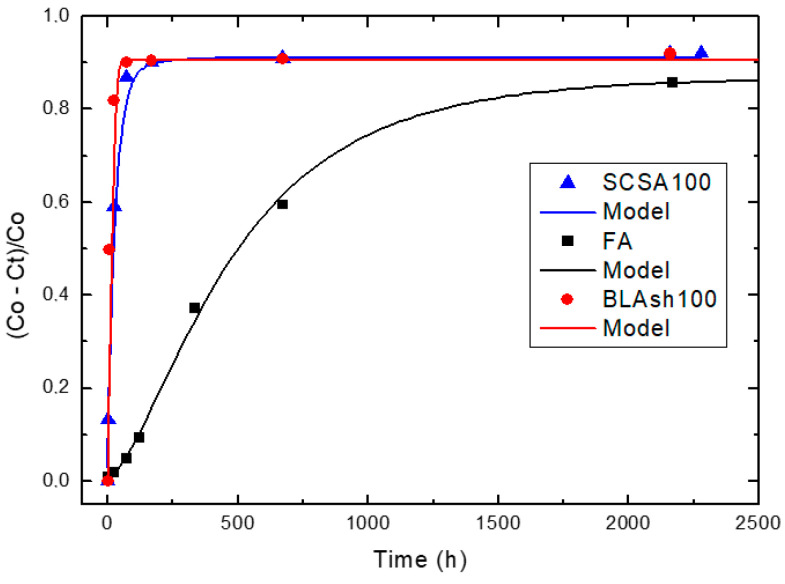
Relative loss of lime concentration versus reaction times for complementary pozzolans SCSA100, FA and BLAsh100 (**Note**: Black circle (experimental), solid line (model)).

Figure 4, Figure 5 and Figure 6 illustrate the relative loss of lime concentration plotted against reaction time for binary systems 1SCBA + BLAsh, 2SCBA + SCSA and PS + FA. Figure 7 shows the samples LS, FA and SCSA that are used as complementary components in the binary mixtures. The solid and dash lines represent the curves of the fitted model.

Fitting the relative loss of lime concentration versus reaction time successively to the model in its different variants (kinetic control, diffusive control and a mixed (kinetic-diffusive control) and analyzing important parameters, such as coefficient of multiple determination (R^2^), residual sum of squares (RSS), 95% confidence intervals, residual scatter, residual probability and variance analysis, it can be stated that:

For the case of SCWI, 2SCBA50SCSA50, SCSA100, 2SCBA100, 1SCBA50BLAsh50, 1SCBA60BLAsh40, 1SCBA70BLAsh30, BLAsh100, PSLSFA and LS the best correspondence with the experimental data is showed by the kinetic control model. This implies that the chemical reaction speed on the surface of the nucleus of the pozzolan particle is slower than the diffusion speed of CH through the reaction product layer formed around the nucleus. This is due to a quick diffusion process, which is facilitated by the high porosity of the reaction product layer in these materials [22].

For the samples of 1SCBA100, PSISFA and FA, the best correspondence with the experimental data is showed by the kinetic-diffusive control model. The regime that predominates is kinetic-diffusive. This means that the diffusion speed of the reactant through the reaction product layer around the nucleus and the chemical interaction speed on the surface of the nucleus of the pozzolan particle are comparable. Therefore, both processes determine the general speed of the whole process.

The values of the parameters (τ, K and *C_corr_*) are given in Table 3.

Taking into account the values of the kinetic parameters, it is possible to conclude that: For the binary system 2SCBA + SCSA, the binary mixture 2SCBA50SCSA50 has the highest reactivity (order of 10^−2^ h^−1^). This reactivity is greater than that of the base pozzolan 2SCBA100 (order of 10^−3^ h^−1^) (the one with the highest availability or most abundance) and less than the complementary pozzolan SCSA100 (order of 10^−1^ h^−1^) (with the lowest availability or minor abundance). The synergy that arises between 2SCBA and SCSA is appreciated because the pozzolanic binary mixture exceeds 2SCBA100. This reactivity was in the same order as the rice husk ashes and sugar cane straw ashes, one order lower than the bamboo leaf ashes, materials considered in the technical literature to be high pozzolanic reactivity ashes [68,69]

The binary mixture SWI showed a minor reactivity (in one order near to 10^−3^ h^−1^).

For the binary system 1SCBA + BLAsh, 1SCBA60BLAsh40 has the highest reactivity (order of 10^−1^ h^−1^). This reactivity is much higher than that of the pozzolan base 1SCBA100 (in an order near to 10^−3^ h^−1^). It is the one with the highest availability or most abundance. This reactivity is also less than the complementary pozzolan BLAsh100 (with the lowest availability or less abundance). The synergy that arises between 1SCBA and BLAsh is appreciated because the reactivity of the pozzolanic binary mixture is more than 1SCBA.

For the binary system PS + FA, PSLSFA has the highest reactivity of the binary system with a very high reactivity (order of 10^−2^ h^−1^). This reactivity is greater than that of the base pozzolan LS (order of 10^−3^ h^−1^) (which exists with greater availability) and also greater than the complementary pozzolan FA (order of 10^−3^ h^−1^) (with less availability). The good synergy that arises between LS and FA is appreciated when the pozzolanic reactivity of the binary mixture is more than its LS and FA components.

Analyzing the three binary systems, we can point out that the 1SCBA + BLAsh system is in general the one that shows the greatest pozzolanic reactivity, showing the highest values of the reaction rate constant K (order of 10^−1^ h^−1^) for all binary mixtures. The highest pozzolanic reactivity is shown by 1SCBA60BLAsh40 (the same order as the silica fume) [69], followed by the PS + FA system, specifically the PSLSFA binary mixture. In the 2SCBA + SCSA system, the 2SCBA50SCSA50 binary mixture shows the highest reactivity.

These results agree with the qualitative analysis carried out previously in Section 2.2 above.

## 4. Conclusions

The conclusions drawn from the present study are listed below:

– Chemically, all the binary samples are formed by the same oxides but with different contents. The main oxides are silica and alumina (although PSLSFA and POISFA shows a 47% and 54% of CaO respectively), whose contents are: 71.6% (SCSA), 70.5% (BLAsh), 69.79% (2SCBA), 60.10% (SCWI), 55.70% (FA), 75.39% (LS), 72.13% (SCSA), 49.79% (2SCBA50SCSA50) and 36.20% (1SCBA).
– The values of the reaction rate constant, obtained in the fitting process of the kinetic-diffusive model, show that the binary blends 1SCBA60BLAsh40 (6.18 · 10^−1^ h^−1^), 1SCBA50BLAsh50 (1.72 · 10^−2^ h^−1^), 1SCBA70BLAsh30 (2.89 · 10^−1^ h^−1^) have a very high pozzolanic reactivity followed by PSLSFA (6.26 · 10^−2^ h^−1^), 2SCBA50SCSA50 (1.72 · 10^−2^ h^−1^), PSISFA (5.51 · 10^−3^ h^−1^) and SCWI (9.32 · 10^−4^ h^−1^).
– Analyzing the three binary systems we can point out that the 1SCBA + BLAsh system is, in general, the one that shows the greatest pozzolanic reactivity, showing the highest values of the reaction rate constant K (order of 10^−1^ h^−1^) for all the binary mixtures, the highest being 1SCBA60BLAsh40 (the same order of silica fume), followed by the PS + FA system, specifically the PSLSFA binary mixture, and by the 2SCBA + SCSA system, specifically the 2SCBA50SCSA50 binary mixture. In all these cases, there is a good synergy between the basic and complementary pozzolans, and the mentioned binary mixtures have greater reactivity than the basic pozzolans (1SCBA, 2SCBA and LS).
– The availability of pozzolanic blends with different pozzolanic reaction rates can become an important technological advantage in the manufacturing of new ternary cements that include pozzolanic binary blends. The selection of one binary system or another as the one preferred will depend on the characteristics needed for the building site.

## Figures and Tables

**Table 1:** Designations of the samples used in the present work.

Pozzolanic Binary Systems	Samples	SCBA (%)	SCSA (%)	FA (%)	BLAsh (%)	Paper Sludge	Designation
Sugar Cane Bagasse Ash and Sugar Cane Straw Ash (2SCBA + SCSA)	2SCBA + SCSA (industrial)	50	50	-	-	-	SCWI
	2SCBA + SCSA (Laboratory)	50	50	-	-	-	2SCBA50SCSA50
	SCSA	-	100	-	-	-	SCSA100
	2SCBA	100	-	-	-	-	2SCBA100
Sugar Cane Bagasse Ash and Bamboo Leaf Ash (1SCBA + BLAsh)	1SCBA + BLAsh	50	-	-	50	-	1SCBA50Blash50
	1SCBA + BLAsh	60	-	-	40	-	1SCBA60Blash40
	1SCBA + BLAsh	70	-	-	30	-	1SCBA70Blash30
	1SCBA	100	-	-	-	-	1SCBA100
	BLAsh	-	-	-	100	-	BLAsh100
Paper sludge (PS) (industrial and laboratory) and Fly Ash (FA) (PS + FA)	Paper sludge (LS) +FA	- -	-	50	-	50	PSLSFA
	Paper sludge (IS) + FA	-	-	50	-	50	PSISFA
	Paper sludge	-	-	-	-	100	PS

**Table 2:** Chemical composition of the binary pozzolanic systems.

	1SCBA/BLAsh System		2SCBA/SCSA System				Paper Sludge/Fly Ash System		
Oxide (%)	1SCBA 100	BLAsh100	2SCBA100	SCSA 100	2SCBA50SCSA50	SCWI	PSLSFA	PSISFA	FA
SiO_2_	36.20	70.5	69.40	71.6	49.79	60.10	13.90	11.02	55.70
Al_2_O_3_	12.30	0.63	11.26	0.58	7.53	12.50	8.30	9.37	24.00
Fe_2_O_3_	8.76	0.47	5.41	0.37	4.43	10.35	0.50	0.72	4.80
CaO	7.10	7.86	2.51	7.47	11.10	3.11	47.12	54.36	2.20
MgO	4.76	1.84	1.28	1.48	7.43	2.10	1.60	1.20	0.90
SO_3_	4.38	2.87	1.83	2.69	1.95	0.10	0.00	0.39	0.89
K_2_O	12.80	5.14	3.45	4.95	8.45	6.00	0.30	0.21	2.18
P_2_O_5_	5.42	1.67	1.61	1.44	2.68	1.47	0.20	0.27	0.28
Na_2_O	0.20	<0.001	0.09	<0.001	0.28	0.16	0.23	0.12	0.45
TiO_2_	1.98	0.06	1.38	0.07	0.86	2.73	0.25	0.50	0.69
LOI	5.37	7.79	1.56	8.08	5.08	1.00	26.66	21.68	7.60

**Table 3:** Reaction rate constant K, *τ* parameter, *C_corr_*. parameter and statistical parameters for the pozzolanic materials.

Pozzolanic Binary Sistems	Material (Ash)	*τ* (h)	Reaction Rate Constant K (h^−1^)	Diffusion Coeffient De (mm^2^/h)	*Ccorr.*	Coefficient of Multiple Determination (R^2^)	Residual Sum of Squares
2SCBA + SCSA	SCWI	152.9 ± 10.3	(9.32 ± 0.23) · 10^−4^		0.07 ± 0.003	0.9775	0.0194
	2SCBA50SCSA50	33.1 ± 2.7	(1.72 ± 0.25) · 10^−2^		0.051 ± 0.038	0.9788	0.0020
	SCSA100	23.2 ± 1.8	(8.12 ± 0.67) · 10^−1^		0.09 ± 0.01	0.9976	0.0022
	2SCBA100	43.5 ± 3.1	(6.91 ± 0.82) · 10^−3^		0.10 ± 0.02	0.9722	0.0125
1SCBA + BLAsh	1SCBA50Blash50	5.6 ± 0.2	(3.83 ± 0.02) · 10^−1^		0.33 ± 0.03	0.9863	0.0620
	1SCBA60Blash40	3.6 ± 0.1	(6.18 ± 0.03) · 10^−1^		0,41± 0.02	0.9780	0.0410
	1SCBA70Blash30	4.5 ± 0.2	(2.89 ± 0.03) · 10^−1^		0.45 ± 0.002	0.9810	0.0420
	1SCBA100	5.3 ± 0.06	(1.85 ± 0.004) · 10^−2^	(1.13 ± 0.005) · 10^−2^	0.74 ± 0.0006	0.9952	0.0032
	BLAsh100	2.9 ± 0.07	(10.02 ± 0.03) · 10^−1^		0.25 ± 0.003	0.9873	0.0570
PS + FA	PSLSFA	93.9 ± 2.07	(6.26 ± 0.90) · 10^−2^		0.13 ± 0.030	0.9810	0.0165
	PSISFA	272.1 ± 31.4	(5.51 ± 0.12) · 10^−3^	(4.71 ± 0.85) · 10^−3^	0.17 ± 0.021	0.9894	0.0072
	LS	50.2 ± 2.28	(8.28 ± 0.09) · 10^−3^		0.30 ± 0.005	0.9979	0.0006
	FA	301.6 ± 28.2	(2.12 ± 0.47) · 10^−3^	(2.21 ± 0.88) · 10^−3^	0.13 ± 0.04	0.9984	0.0012

## Data Availability

Data sharing not applicable.
